# DNA Damage Baseline Predicts Resilience to Space Radiation and Radiotherapy

**DOI:** 10.1016/j.celrep.2020.108434

**Published:** 2020-11-25

**Authors:** Eloise Pariset, Antonella Bertucci, Margaux Petay, Sherina Malkani, Alejandra Lopez Macha, Ivan G. Paulino Lima, Vanesa Gomez Gonzalez, Antony S. Tin, Jonathan Tang, Ianik Plante, Egle Cekanaviciute, Marcelo Vazquez, Sylvain V. Costes

**Affiliations:** 1 NASA Ames Research Center, Space Biosciences, Moffett Field, CA 94035, USA; 2 Universities Space Research Association (USRA), Mountain View, CA 94043, USA; 3 Loma Linda University, Loma Linda, CA 92350, USA; 4 Blue Marble Space Institute of Science, Seattle, WA 98154, USA; 5 Exogen Biotechnology, Berkeley, CA 94710, USA; 6 KBR, NASA Johnson Space Center, Houston, TX 77058, USA; 7 Lead Contact

## Abstract

Deep space exploration will require real-time, minimally invasive monitoring of astronaut health to mitigate the potential health impairments caused by space radiation and microgravity. Genotoxic stress in humans can be monitored by quantifying the amount of DNA double-strand breaks (DSBs) in immune cells from a simple finger prick. In a cohort of 674 healthy donors, we show that the endogenous level of DSBs increases with age and with latent cytomegalovirus infection. To map the range of human responses to space radiation, we then study DSB induction and repair in immune cells from 319 healthy donors after the cells are exposed to galactic cosmic ray components and lymphocytes from 30 cancer patients after radiotherapy. Individuals with low baseline DSB have fewer clinical complications, enhanced DNA damage repair responses, and a functional dose-dependent cytokine response in healthy donor cells. This supports the use of DSB monitoring for health resilience in space.

## In Brief

Pariset et al. find that individuals with a lower baseline level of DNA damage are resilient to clinical complications after radiotherapy and present higher radiation-induced levels of DNA repair foci and inflammatory cytokines. These findings support the use of baseline DNA damage as a biomarker for radiation sensitivity.

## Graphical Abstract

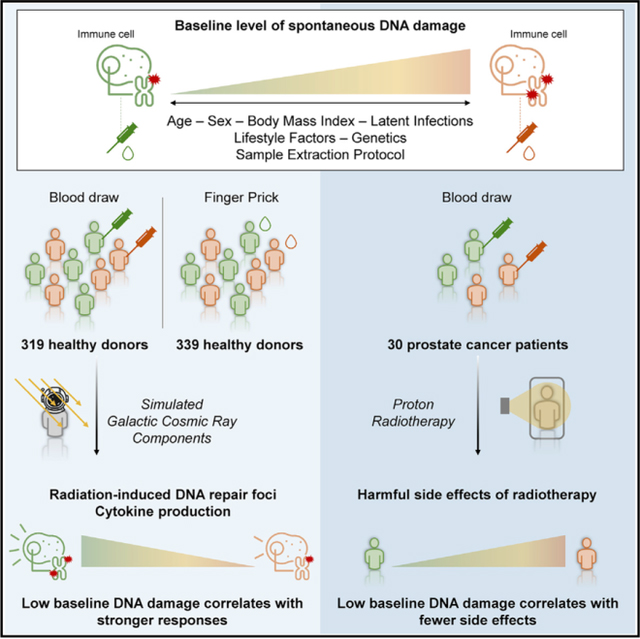

## INTRODUCTION

Exposure to ionizing radiation is a major spaceflight-associated health risk in both low-Earth orbit and deep space beyond the protective magnetic field of the Earth. Combining in-flight environmental telemetry with constant health monitoring will be crucial for predicting and counteracting potential detrimental outcomes to individual astronauts. Similarly, on Earth, individual resilience to DNA damage can be used in cancer radiotherapy to triage patients toward more efficient therapies. For example, radiation-resilient patients could be able to sustain harsher hypofractionation protocols that cause heightened inflammation (Paul-Gilloteaux et al., 2017) but provide better tumor control and faster treatment by delivering few high-dose fractions.

Exposure to high-linear energy transfer (LET) particle radiation is a particularly important health risk that will be experienced beyond low-Earth orbit (Nelson, 2016). The main health risks of space radiation exposure include carcinogenesis, immune dysfunction, cardiovascular damage, and central nervous system deficits (Cucinotta et al., 2009; Huff et al., 2016; Krukowski et al., 2018; Kumar et al., 2018; Patel et al., 2016). Increased susceptibility to infections, reactivation of latent viruses, and systemic immune dysregulation have been observed in astronauts (Crucian et al., 2008, 2015; Mehta et al., 2017). Radiation largely contributes to this response, because circulating immune cells, especially lymphocytes, are among the most radiosensitive cell types in the human body (Nosel et al., 2013). Such sensitivity can be exploited for developing better health monitoring, especially because blood is a relatively easily accessible cell source.

Effects of ionizing radiation include both direct DNA damage and indirect cellular oxidative stress responses caused by elevated reactive oxygen species (ROS) and reactive nitrogen species. DNA double-strand breaks (DSBs) are recognized by DNA damage-sensing proteins that form nuclear domains called radiation-induced foci (RIFs) (Costes et al., 2006; Rogakou et al., 1998). RIF measurements are routinely used to quantify DSBs and evaluate DNA repair kinetics after either space or medical radiation exposure (Costes et al., 2010; Roos et al., 2016). A standard metric to quantify RIF formation is immunostaining for markers of DNA repair proteins, such as tumor suppressor 53 binding protein-1 (53BP1) (Georgescu et al., 2015).

The indirect effects of ionizing radiation are primarily mediated by elevated ROS, which are produced during the radiolysis of water (Cadet et al., 1999). They can lead to self-perpetuating oxidative stress, which contributes to inflammation at the tissue and organ levels, further exacerbating DNA damage and eventually leading to cellular damage and cell death (Azzam et al., 2012; Maxwell et al., 2008; Paul et al., 2020). Thus, direct and indirect effects of deep space radiation must be considered simultaneously when assessing the impact on health.

In this study, we report that baseline DNA damage levels in human peripheral blood mononuclear cells (PBMCs), as measured by 53BP1^+^ foci quantification before irradiation, can predict direct and indirect *ex vivo* responses to simulated space radiation exposure, as well as toxicity outcomes from cancer radiotherapy treatments. To perform this work, we have developed a semi-automated, high-throughput method to quantify DNA damage and repair based on 53BP1 immunocytochemistry of PBMC subtypes and applied it to immune cells isolated from healthy donors and cancer patients using a venous blood draw or a finger prick.

Our results indicate that human immune responses to simulated deep space radiation and to therapeutic radiation are correlated with levels of baseline DNA damage; individuals with low baseline levels of DNA damage were more resilient, possibly because of a protective feedback mechanism. More generally, this work supports the use of PBMCs and DNA damage-sensing protein 53BP1 expression as a minimally invasive astronaut monitoring tool to identify and to anticipate potential health impairments during long-term spaceflight from a simple finger prick, as well as a potential predictive metric of cellular damage for cancer radiotherapy.

## RESULTS

### 53BP1 Is a Robust DNA Damage Marker for Foci Detection

**Figure 1.**
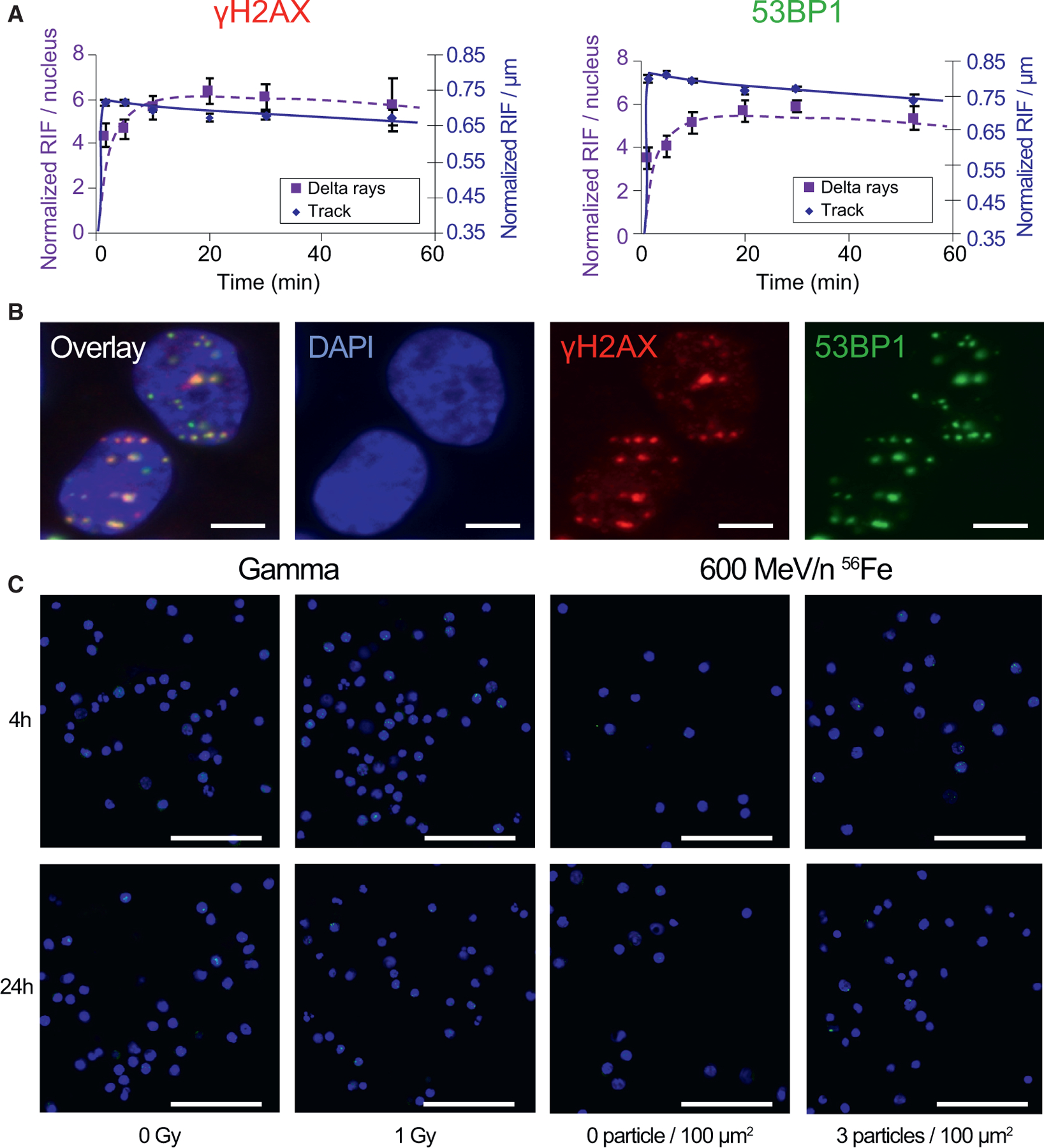
53BP1 Is a Robust DNA Damage Marker for Foci Detection (A) Representative time response of background corrected γH2AX (left) and 53BP1 (right) RIFs per nucleus in MCF10A exposed to 1 Gy of 1 GeV/n ^56^Fe ions. (B) Representative image of two MCF10A nuclei at 30 min post-^56^Fe irradiation in overlay, DAPI, γH2AX, and 53BP1 channels, illustrating the co-localization of both RIF markers. Scale bar: 5 µm. (C) Representative images of PBMC nuclei (DAPI) and 53BP1^+^ foci (fluorescein isothiocyanate [FITC]) at 4 and 24 h after 1 Gy of gamma rays or 3 particles/100 µm^2^ (0.82 Gy) of 600 MeV/n ^56^Fe ions and nonirradiated controls. Scale bar: 50 µm.

We first validated our method of foci quantification using nonmalignant human mammary epithelial cells (MCF10A) exposed to 1 Gy of 1 GeV/n ^56^Fe irradiation. The distribution of 53BP1 RIF was compared with the distribution of γH2AX RIFs in the same MCF10A cells between 0 and 60 min post-irradiation. Figure 1A represents the number of γH2AX and 53BP1 RIFs normalized to the number of foci in unirradiated controls and classified as core RIFs or delta-ray-induced RIFs based on their radial distance from the tracks of the irradiation particles (Neumaier et al., 2012). Similar time responses were observed for γH2AX and 53BP1 RIF formation and repair, with fast RIF induction along the tracks and slower RIF induction in the regions in which delta rays radiate from the tracks. In addition, the number of γH2AX and 53BP1 RIFs was similar for delta-ray-induced RIFs, whereas 53BP1 detection was more sensitive to core RIFs. Figure 1B confirms the co-localization of γH2AX and 53BP1 RIFs at 30 min post-^56^Fe irradiation.

In human PBMCs, 53BP1 foci were successfully detected in DAPI-stained nuclei, with increased levels of foci after exposure to 600 MeV/n ^56^Fe and decreased RIF numbers at 24 h compared with 4 h post-irradiation (Figure 1C).

### Baseline Level of Spontaneous DNA Damage Is Affected by Demographic Variability

**Figure 2.**
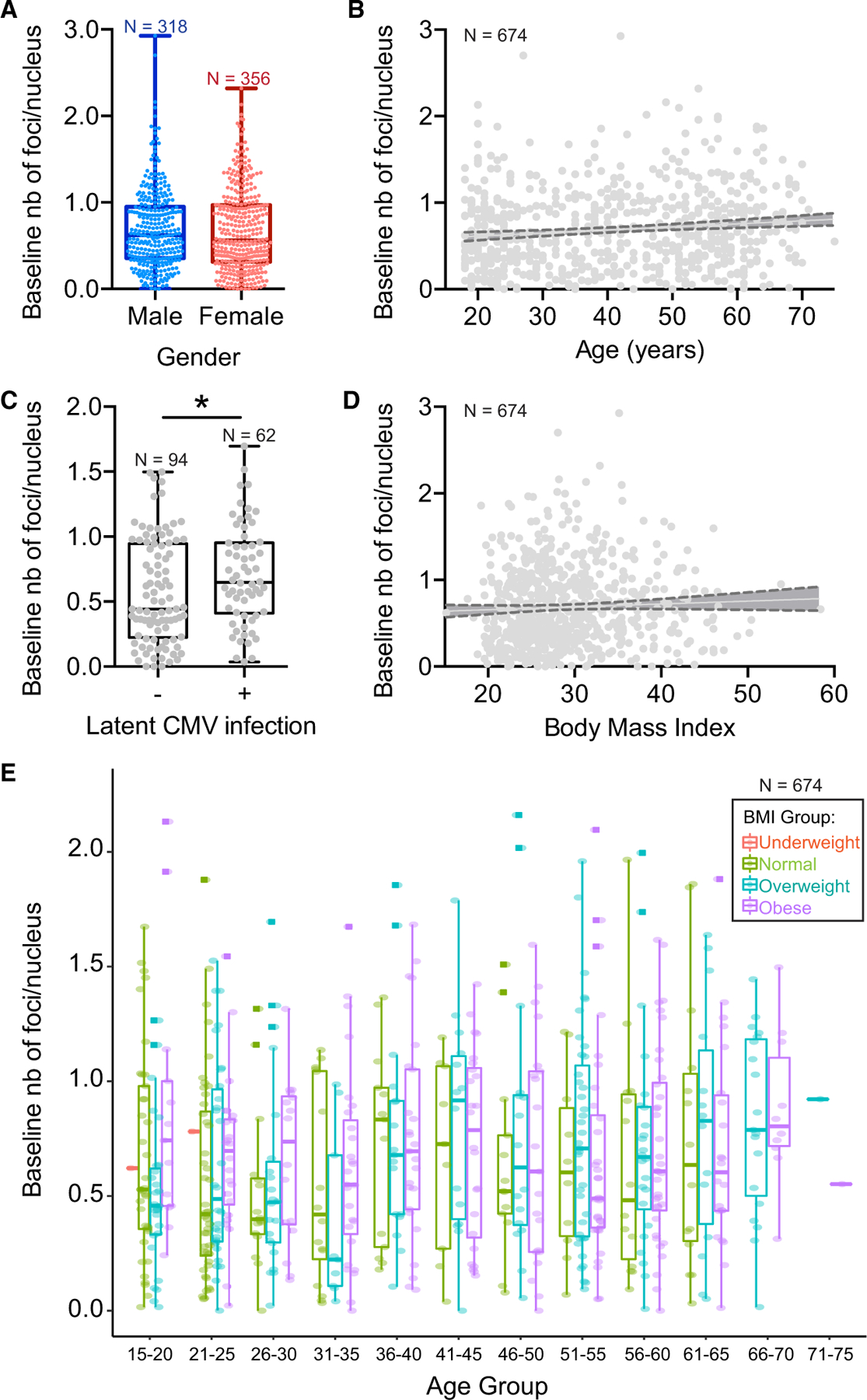
Baseline Level of Spontaneous DNA Damage in PBMCs Is Affected by Demographic Variability Distribution of baseline number of foci per nucleus in PBMCs as a function of demographic variables. (A) Gender (p = 0.56, unpaired t test with Welch’s correction). (B) Age (**p = 0.0019, deviation from zero of the slope of linear regression). See also Figure S1. (C) Latent cytomegalovirus (CMV) infection (*p = 0.036, unpaired t test with Welch’s correction). (D) Body mass index (BMI) (p = 0.239, deviation from zero of the slope of linear regression). (E) Age and BMI groups (interaction of age and BMI, p = 0.39). The dark gray areas in (C) and (D) represent the 95% confidence interval on the slope of the linear regression model. Boxplots show median ± quartiles ± min/max values.

We quantified the baseline level of spontaneous DNA damage in PBMCs from 674 healthy donors (47%/53% male/female, 18–75 years old) to evaluate inter-individual variability and identify key demographic factors associated with baseline foci levels. Baseline DNA damage was measured using immunocytochemistry for DNA repair protein 53BP1 followed by high-throughput automated detection of 53BP1^+^ intra-nuclear foci in PBMCs. Figure 2 represents the amount of baseline DNA damage as a function of gender (Figure 2A), age (Figure 2B), latent cytomegalovirus (CMV) infection (Figure 2C), and body mass index (BMI) (Figure 2D).

A high variability of baseline DNA damage was observed across subjects, ranging from 0 to 3 foci/nucleus, with a median of 0.6 foci/nucleus (Figure 2). Although sex had no effect on baseline levels of DNA damage (Figure 2A, mean of 0.70 and 0.68 foci/cell in males and females, respectively), we observed a significant increase in baseline DNA damage with age based on the slope of the linear regression model on individual data (slope significantly different from zero at **p = 0.0019, Figure 2B), as well as one-way ANOVA between 10-year age groups (**p = 0.0093, Figure S1A). There was no difference between BMI groups across all individuals (Figures 2D and E). Although all donors were screened to eliminate individuals with a history of neurological disorders, autoimmune disease, cancer, and infections, it is likely that several lifestyle factors, such as smoking habits, alcohol consumption, diet, and exercise, as well as genetic and epigenetic factors, contribute to the important inter-individual variabilities observed in baseline levels of DNA damage.

We analyzed the latent viral infection status of our donors because of their potential connections to autoimmune diseases and peripheral blood immune cell distribution. Although all donors were negative for Zika, Chagas, HTLV (Human T cell Lymphotropic Virus), HCV (Hepatitis C Virus), HIV, and West Nile virus infections, significantly higher levels of baseline DNA damage were observed in individuals who tested positive for latent CMV infection as opposed to CMV-negative donors (Figure 2C, mean of 0.57 and 0.70 foci/cell in CMV-negative and CMV-positive donors, respectively). The increased level of baseline DNA damage in CMV^+^ donors compared with CMV^−^ donors suggests the possibility that a latent viral infection may sensitize PBMCs to other stressors, including DNA damage, lowering the threshold for induction of DNA repair mechanisms. Alternatively, the infection might be associated with a change in immune cell distribution, increasing the proportion of immune cells that are more likely to undergo DNA damage in response to baseline environmental stressors.

**Figure 3.**
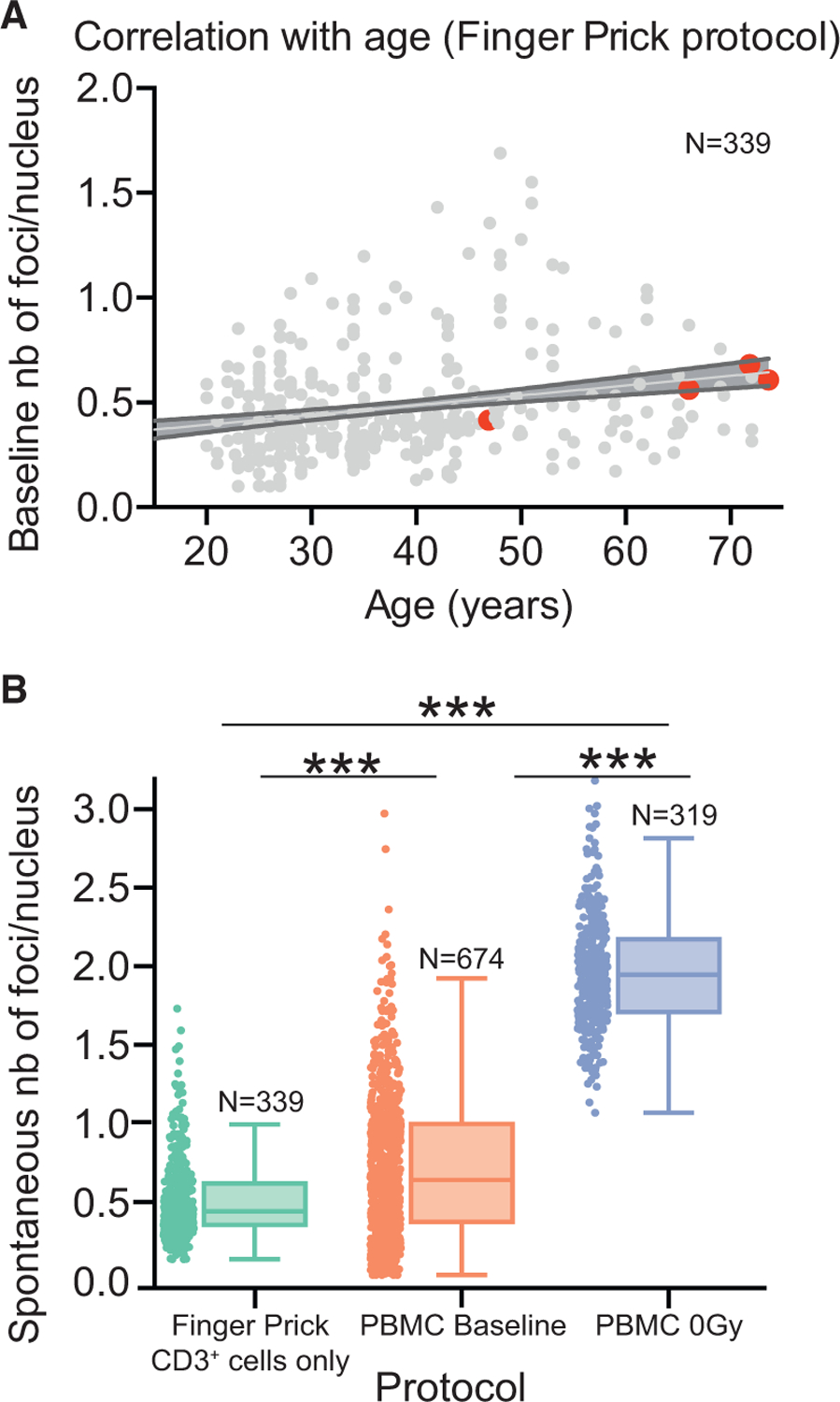
Baseline Level of Spontaneous DNA Damage Increases with Age and Is Affected by Sample Processing (A) Baseline number of foci per nucleus as a function of age in CD3^+^ lymphocytes extracted from finger prick collection in 339 healthy donors (***p < 0.0001, deviation from zero of the slope of linear regression). Four donors in this cohort were considered healthy but were in remission from cancer at the time of the finger prick (shown as red dots). See also Figure S1. (B) Distribution of spontaneous foci per nucleus after bead-based extraction of CD3^+^ lymphocytes from finger prick samples (finger prick), Ficoll-based extraction of PBMCs from buffy coat samples (PBMC baseline), and a further freeze-thaw cycle and plating in the cell culture media (PBMC 0 Gy). ***p < 0.0001, one-way ANOVA, Tukey’s post hoc test for multiple comparisons among all three groups: 1–2, 2–3, and 1–3. Boxplots show median ± quartiles ± min/max values.

To validate DNA damage dependence on age, an independent cohort of 339 healthy donors was evaluated for baseline DNA damage. This time, only age and cancer history metadata were collected. For this, CD3^+^ lymphocytes (T cells) were isolated by magnetic separation from blood samples collected via finger prick, instead of all PBMC obtained via Ficoll-based extraction from regular blood draw, as shown in Figure 2. Similar to Figure 2B, a significant increase in baseline DNA damage was observed with age (slope of the linear regression model significantly different from zero at ***p < 0.0001, Figure 3A, and one-way ANOVA between 10-year age groups at ***p < 0.0001, Figure S1B) in the PBMC subpopulation despite using a different collection protocol. Linear models for the evolution of baseline DNA damage with age gave similar ranges of slope values within the 95% confidence interval for both healthy cohorts: 1.3.10^−3^ to 5.8.10^−3^ foci/nucleus/year for the cohort of 674 donors and 2.7.10^−3^ to 6.7.10^−3^ foci/nucleus/year for the cohort of 339 donors.

Although the trend of increased spontaneous DNA damage with age was observed independently of the PBMC extraction protocol, different protocols significantly affected the median value of spontaneous DNA damage (Figure 3B). One interpretation could be that the more PBMC samples are processed, the more additional background DNA damage is observed. Indeed, extracting PBMCs from blood (Figure 3B, PBMC baseline) induces both more DSBs and more inter-individual variation. Once cells are frozen, thawed, and plated in culture media (Figure 3B, PMBC 0 Gy), even more DSBs are observed, but the variance among individuals is reduced, possibly because the potential confounding factors from the blood microenvironment are removed and the remaining influences on variance are limited primarily to genetic factors. The different cell-subtype composition resulting from the bead-based and the Ficoll-based protocols could be another reason for the different distributions of background DNA damage levels between finger prick and PBMC baseline samples.

The proposed finger prick protocol appears to be particularly robust because of the limited processing steps applied to each sample before fixation, providing the strongest evidence that baseline DNA damage increases with age and the best application for health monitoring in space. Thus, we applied the same method of fixation and lymphocyte extraction to evaluate whether the level of baseline DNA damage can predict clinical outcomes in prostate cancer patients.

### High Baseline DNA Damage in T Cells Results in More Severe Clinical Secondary Effects after Proton Radiotherapy

**Figure 4.**
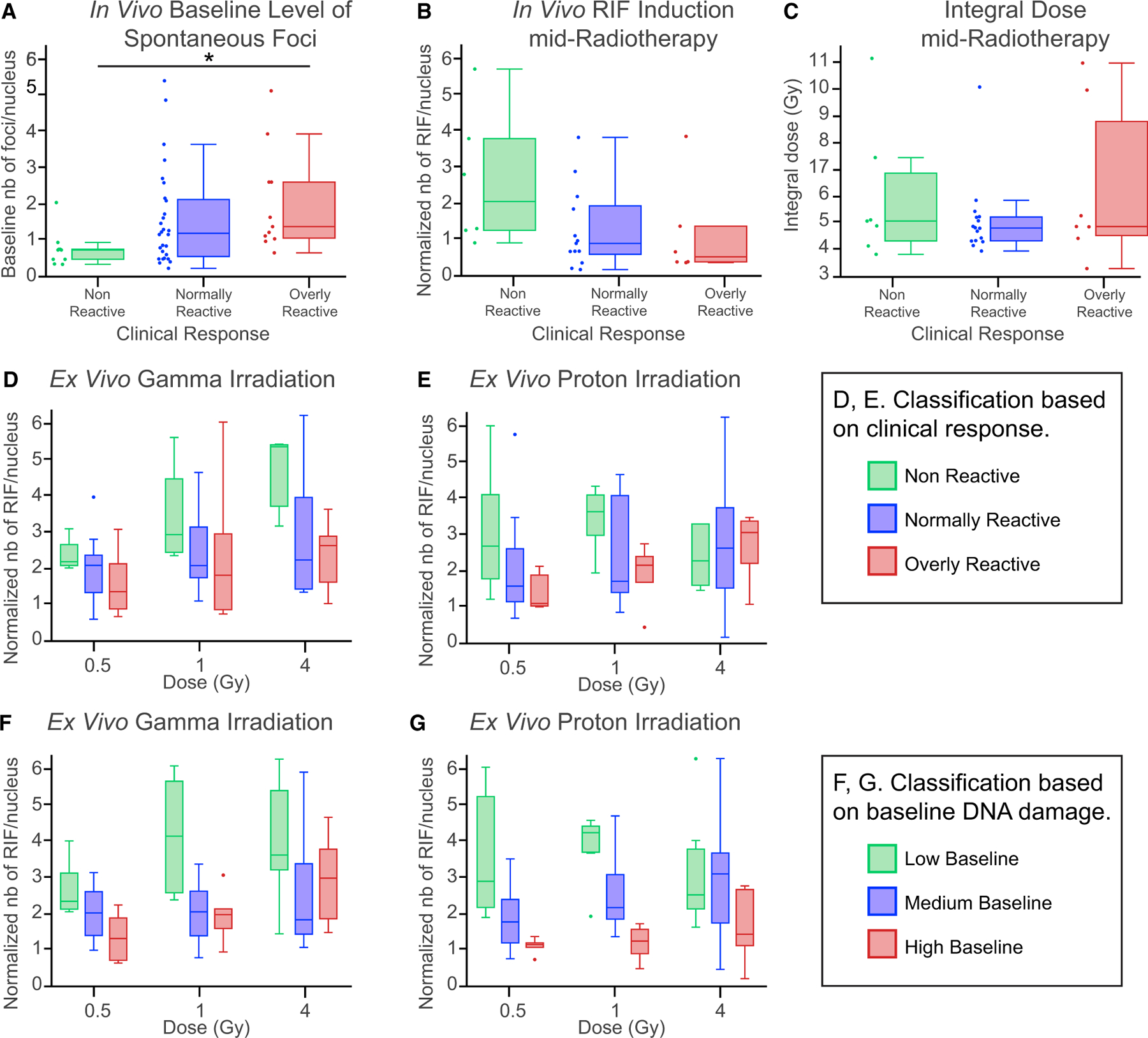
High Baseline DNA Damage in T Cells Results in More Severe Clinical Secondary Effects after Proton Radiotherapy (A) Distribution of baseline number of foci per nucleus for each group of patients classified based on clinical secondary effects to proton radiotherapy. N = 6 nonreactive (NoR), 15 normally reactive (NR), and 7 overly reactive (OR). *p = 0.069, one-way ANOVA. (B) Normalized number of radiotherapy-induced RIFs per nucleus at 1 month of treatment: N = 6 NoR, 13 NR, and 6 OR. p = 0.119, one-way ANOVA. (C) Total pelvic dose received at mid-treatment: N = 7 NoR, 16 NR, and 7 OR. p = 0.443, one-way ANOVA. (D–G) Normalized number of RIF per nucleus quantified at 24 h following *in vitro* gamma irradiation (D and F) and proton irradiation (E and G) at doses of 0.5, 1, and 4 Gy with groups classified based on clinical response (D and E) and baseline DNA damage (F and G). Low and high baselines were defined as the first and fourth quartiles, respectively, from the full cohort of 30 patients. The medium baseline corresponds to all other patients. (D) N = 4 NoR-11 NR-5 OR (0.5 Gy), 4-12-4 (1 Gy), and 3-13-5 (4 Gy). *p (dose) = 0.020, *p (group) = 0.032, p (interaction) = 0.46, two-way ANOVA. (E) N = 5-15-6 (0.5 Gy), 5-15-5 (1 Gy), and 6-15-5 (4 Gy). p (dose) = 0.13, *p (group) = 0.069, p (interaction) = 0.49, two-way ANOVA. (F) N = 6-8-6 (0.5 Gy), 6-9-6 (1 Gy), and 6-9-6 (4 Gy). (G) N = 5-15-6 (0.5 Gy), 5-15-5 (1 Gy), and 6-15-5 (4 Gy). *p (dose) = 0.074, ***p (group) < 0.001, *p (interaction) = 0.072, two-way ANOVA. Boxplots show median ± quartiles ± min/max values.

Baseline levels of spontaneous foci were evaluated in T cells from 30 prostate cancer patients before the first fraction of proton treatment (Figure 4A). Patients were assigned to one of three categories based on the clinical secondary effect outcomes at the end of the proton therapy: nonreactive, normally reactive, and overly reactive (see Method Details). When the same fixation and isolation protocol was applied (Figure 3B, finger prick), the median baseline DNA damage was higher for all three categories of patients (0.76, 1.19, and 1.41 foci/nucleus for nonreactive, normally reactive, and overly reactive patients, respectively), compared with healthy donors of the same age range (0.55 foci/nucleus for 62- to 78-year-old donors). Interestingly, a significantly higher level of baseline DNA damage was associated with increased severity of clinical secondary effects (Figure 4A). This suggests overly reactive patients who have comparatively dangerous secondary effects from radiation treatment could be detected based on their baseline levels of foci, which is an essential step toward personalizing the radiation treatment to maximize efficacy while minimizing risk.

In addition to baseline DNA damage, the amount of foci induced by proton therapy was evaluated in 25 of the 30 patients at the treatment mid-point (1 month from the first proton treatment fraction). Figure 4B represents the number of RIFs per nucleus normalized by the ratio to the baseline level of foci of the same patient. Though not significant, lower levels of RIFs at mid-radiotherapy were associated with increasing severity of clinical secondary effects (Figure 4B), which does not result from variability in the integral dose received in the pelvic region (Figure 4C). Because RIF levels were quantified based on detection of 53BP1 DNA repair protein, it can be hypothesized that patients with more secondary effects after radiotherapy are less efficient at recruiting 53BP1 at sites of DNA damage, which could alter their DNA damage response.

This observation was supported by *in vitro* irradiation of lymphocytes from all 30 patients. Using the same methods used to assess the baseline levels of DNA damage presented in Figure 4A, RIF levels were quantified in T lymphocytes 24 h after gamma or proton irradiation. Three doses were tested for each irradiation type (0.5, 1, and 4 Gy), and the number of RIFs per nucleus was normalized to the corresponding sham-irradiated control (Figures 4D–4G). For each dose of gamma or proton irradiation, the normalized level of RIFs was again found to anti-correlate with both the severity of clinical secondary effects (Figures 4D and 4E) and the baseline level of DNA damage (Figures 4F and 4G). As expected, an overall increase of RIFs was observed with the dose. The strongest dose dependence was seen for patients with no secondary effects and minimal levels of baseline DNA damage, suggesting these patients might have the most efficient DNA repair process.

However, proton irradiation induced a flattening dose response between 1 and 4 Gy across all groups, which was not observed with gamma irradiation. One possible interpretation of the reduction in RIFs per gray at high dose is the clustering of multiple DSBs into one RIF, as previously described by our group (Costes et al., 2007; Neumaier et al., 2012). Unlike gamma rays, protons generate nearby DSBs along their linear tracks of energy deposition, which leads to higher linear density of DSBs and thus higher probability of fewer RIFs that contain more DSBs per RIF. This interpretation is supported by the saturation effect being stronger in cells from patients with the best DNA repair response (i.e., low baseline and nonreactive patients).

In addition, the flat dose response at a high dose may occur because of cell death triggered by extreme levels of DNA damage in cells showing the highest response to radiation. It has been previously shown that protons have a relative biological effectiveness for cell death larger than 1 (Maks et al., 2011; Mara et al., 2020). This elevated response to radiation in terms of RIF induction and subsequent cell death seems to be beneficial in terms of clinical outcomes to radiotherapy, because patients classified as nonreactive also had the highest RIF levels and the lowest baseline foci levels.

### High Baseline DNA Damage Results in Minimal DNA Damage Response to Galactic Cosmic Ray (GCR) Components

**Figure 5.**
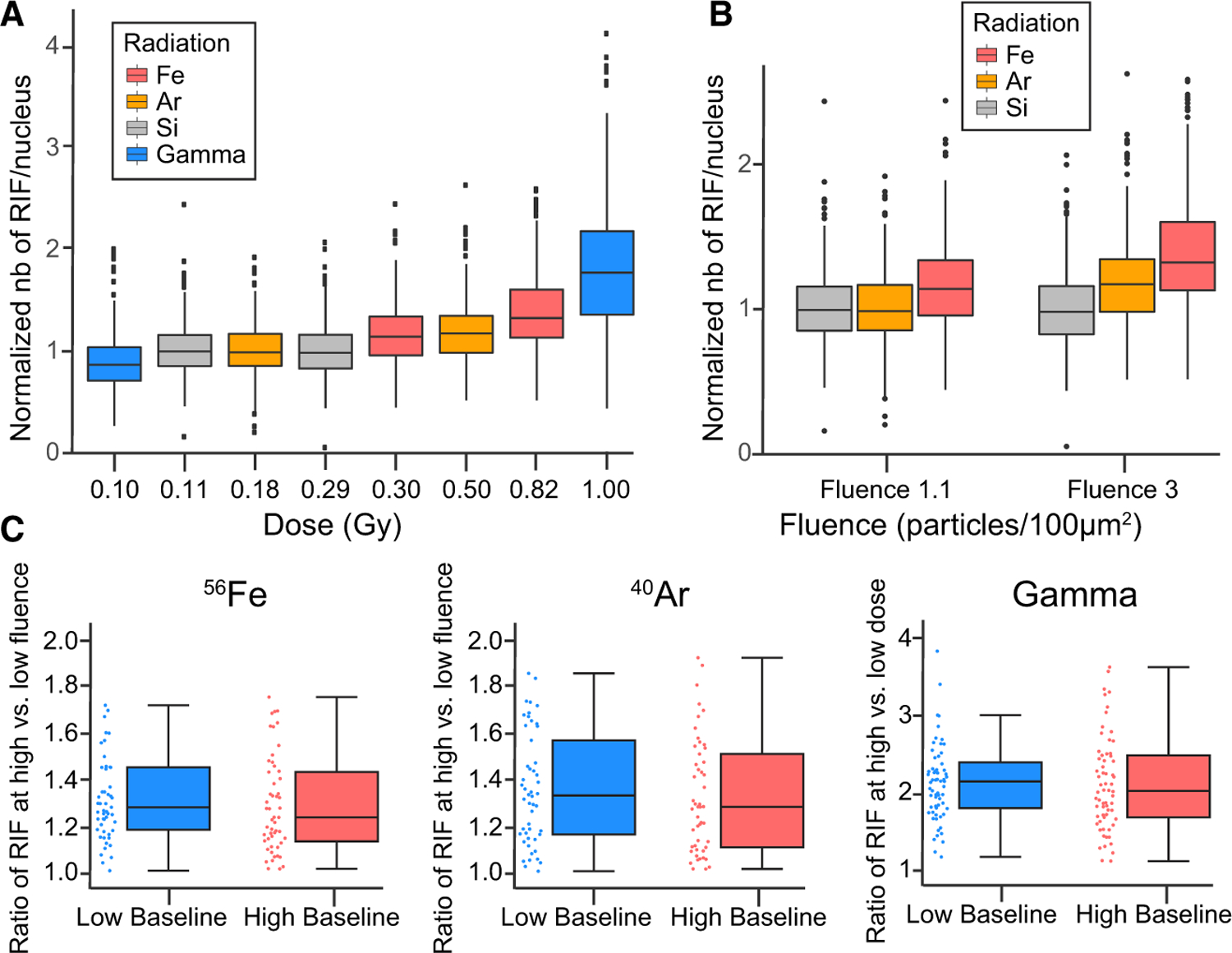
High Baseline DNA Damage Results in Minimal DNA Damage Response to GCR Components (A and B) Normalized number of RIFs per nucleus based on dose (A) or fluence (B) in PBMCs from 319 healthy donors at 4 h after *in vitro* exposure to 0.1 or 1 Gy gamma rays (blue), 0.11 Gy (1.1 particles/100 µm^2^) or 0.29 Gy (3 particles/100 µm^2^) ^28^Si (gray), 0.18 Gy (1.1 particles/100 µm^2^) or 0.50 Gy (3 particles/100 µm^2^) ^40^Ar (orange), and 0.30 Gy (1.1 particles/100 µm^2^) or 0.82 Gy (3 particles/100 µm^2^) ^56^Fe (red). (A) ***p < 0.001, one-way ANOVA. (C) High:low fluence ratio of RIFs for ^56^Fe and ^40^Ar irradiation and high:low dose ratio of RIFs for gamma irradiation for each group of donors, separated based on baseline level of RIFs (N = 53 donors in each group for ^56^Fe, N = 50 donors each for ^40^Ar, and N = 65 donors each for gamma). ^56^Fe, p = 0.13; ^40^Ar, p = 0.51; gamma, p = 0.54; t test with Welch’s correction. Boxplots show median ± quartiles ± min/max values.

The most harmful components of GCR, to which astronauts will be exposed on missions beyond low-Earth orbit, are high-LET high-mass and high charge and energy (HZE) particles. To determine whether the level of baseline DNA damage is associated not only with responses to therapeutic radiation but also to simulated deep space radiation, we investigated the DNA damage response of PBMCs isolated from 319 healthy donors (whose baseline and 0 Gy control foci levels were shown in Figure 3B) after exposure to the three main HZE particles that compose GCR—350 MeV/n ^28^Si (63 keV/µm), 350 MeV/n ^40^Ar (104 keV/µm), and 600 MeV/n ^56^Fe (170 keV/µm LET)—as well as after exposure to low-LET gamma ray control. Two fluences—1.1 and 3 particles/100 µm^2^—were tested for all particles, corresponding to doses of 0.11 and 0.29 Gy for ^28^Si, 0.18 and 0.50 Gy for ^40^Ar, and 0.30 and 0.82 Gy for ^56^Fe. Similarly, two doses were tested for gamma irradiation: 0.1 and 1 Gy. 53BP1^+^ RIFs were quantified 4 h after irradiation and normalized as fold over nonirradiated control foci levels (Figures 5A and 5B). The expected dose dependence is observed for the higher doses, with a median RIF level increasing 1.3 times for a 1.2-fold increase in dose from 0.82 to 1 Gy. However, only a 1.1-fold RIF increase is induced from 0.5 to 0.82 Gy and a flat dose response is observed below 0.3 Gy, probably due to detection limitations.

Because cancer patients with low levels of baseline DNA damage had higher RIF levels after irradiation, we subselected healthy donors with the most extreme baseline levels of foci to identify whether these individuals also had different RIF responses to high-LET HZE particles. Individuals with low or high baseline levels of DNA damage were selected from the full cohort of 319 healthy donors based on the first and the last quartiles of baseline foci values, respectively. Their RIF levels were evaluated at each of the two doses tested for ^56^Fe, ^40^Ar, and gamma rays (^28^Si responses were omitted, because the tested doses of ^28^Si did not elicit a dose response). The ratio of RIFs at high versus low dose is represented in Figure 5C for both baseline groups. Although not significant, more RIFs were induced with dose in low baseline individuals compared with high baseline individuals (median low baseline to high baseline: 1.28–1.23 for ^56^Fe, 1.33–1.28 for ^40^Ar, and 2.15–2.02 for gamma). The differential between low and high dose observed here was lower than the differential for the whole-blood irradiation response observed with cancer patients, reflecting the importance of the blood microenvironment (e.g., plasma). For instance, gamma irradiation was performed both on whole blood and on isolated cells, inducing a significant differential in the case of whole blood (Figure 4F). This suggests that the microenvironment of the blood is a major factor driving the DNA damage baseline and its relationship to the systemic long-term radiation response outcome.

### Higher Baseline Levels of DNA Damage Correlate with Lower Oxidative Stress Response to GCR Components

**Figure 6.**
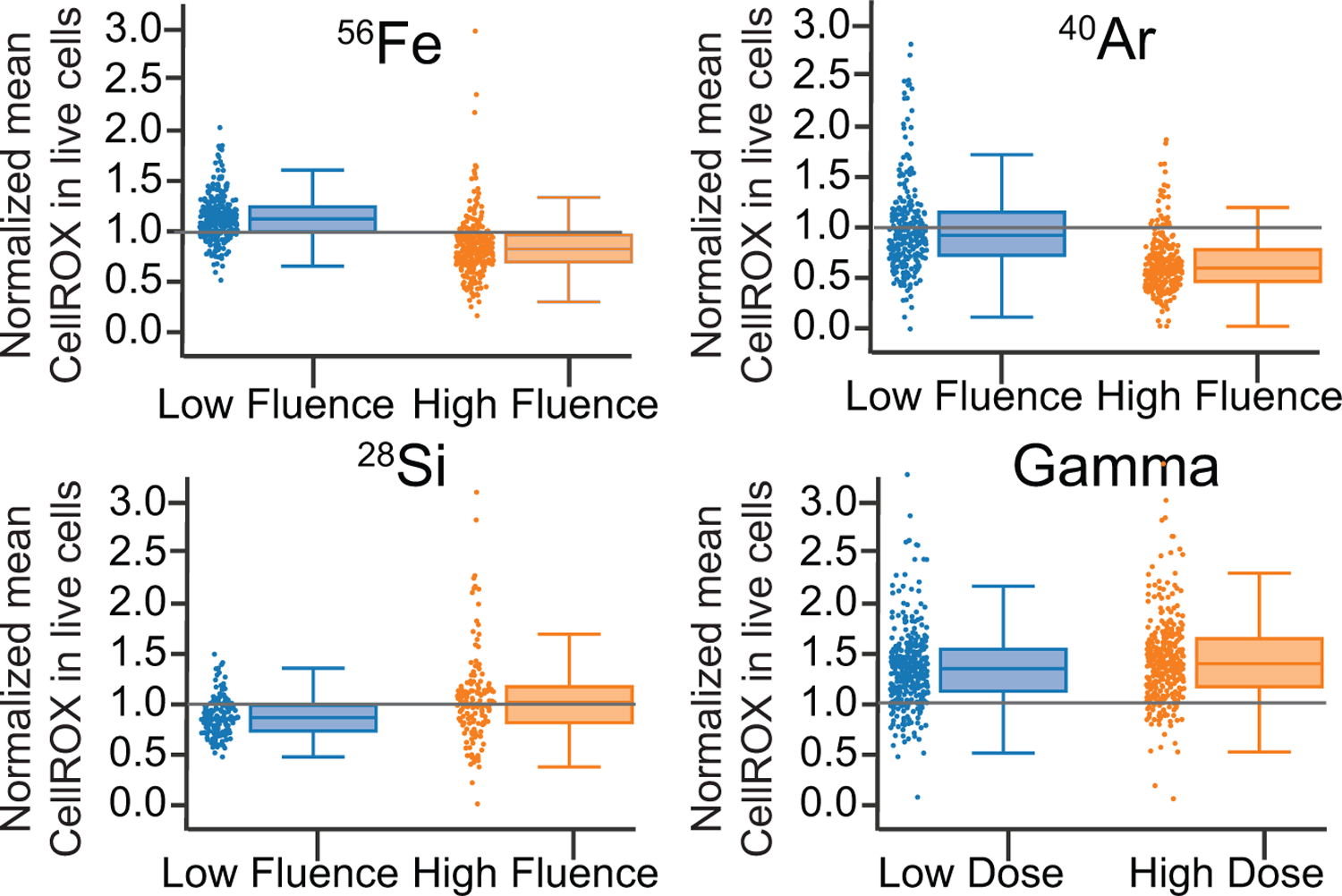
Irradiation with High-LET GCR Particles Reduces Oxidative Stress in Human PBMCs Normalized mean of CellROX quantified at 4 h post-irradiation in live PBMCs from healthy donors following *in vitro* exposure to 1.1 and 3 particles/100 µm^2^ of ^56^Fe (N = 258), ^40^Ar (N = 247), and ^28^Si (N = 143) and 0.1 and 1 Gy of gamma rays (N = 315). Gray lines indicate the control level of CellROX in sham-irradiated samples. ***p < 0.001, significant decrease of mean CellROX at high versus low fluence for ^56^Fe and ^40^Ar; ***p < 0.001, significant increase of mean CellROX at high versus low fluence for ^28^Si; p = 0.89, no significant changes between low and high dose for gamma (p = 0.89); Welch two-sample t test. Boxplots show median ± quartiles ± min/max values.

For a more complete understanding of high-LET cellular effects, we studied indirect oxidative stress, in addition to direct DNA damage, in PBMCs from the same 319 healthy donors. Results were determined by assessing radiation-induced CellROX fluorescence in live cells at 4 h after irradiation and comparing the fluorescence values in sham-irradiated controls (Figure 6). Values above 1 correspond to a radiation-induced increase in oxidative stress, whereas values below 1 suggest a decrease in oxidative stress after irradiation.

For all three particles, the low dose considered did not elicit a statistically significant change in CellROX fluorescence compared with sham-irradiated levels. For the highest fluence, at 3 particles/100 µm^2^, both ^56^Fe and ^40^Ar induced a significant reduction of CellROX fluorescence, whereas no change was seen with ^28^Si. In contrast, both doses of gamma rays significantly increased CellROX levels compared with 0 Gy, which probably reflects distinct radiochemistry.

This LET-dependent oxidative stress response may be partially explained: as radiation LET is reduced, the amount of hydroxyl radicals (·OH) per equal dose increases. By taking into account the radiation track structure, we have computed the concentration of radicals produced per gray of radiation (Table S1). Based on calculations with the code RITRACKS (Relativistic Ion Tracks) (Plante and Devroye, 2017), we observe that the ratio of ·OH to hydrogen peroxide (H_2_O_2_) decreases with the LET, consistent with our observed decrease in oxidative stress responses.

When the LET of the radiation track increases, radicals (especially ·OH) form nearby, which results in a larger probability of chemical reactions (Frongillo et al., 1998). One reaction of particular interest is the recombination of ·OH into H_2_O_2_: ·OH + ·OH → H_2_O_2_. ·OH takes part in many biochemical reactions occurring naturally in cells but because of high reactivity can also damage most macromolecules, including lipids, proteins, and amino acids (Collin, 2019). The peroxidation of lipids is an important example of a chain reaction involving ·OH. H_2_O_2_ is less reactive but is involved in many homeostatic reactions within cells and acts as a molecular mediator of cellular signaling (Di Marzo et al., 2018). Thus, we hypothesize that ·OH and H_2_O_2_ have a distinct effect on oxidative stress, at least as reflected by CellROX fluorescence, primarily reflecting ·OH levels and being countered by a feedback signaling response elicited by higher H_2_O_2_ levels.

### Higher Baseline Levels of DNA Damage Correlate with Lower Expression of Immune Cytokines after Irradiation with GCR Components

**Figure 7.**
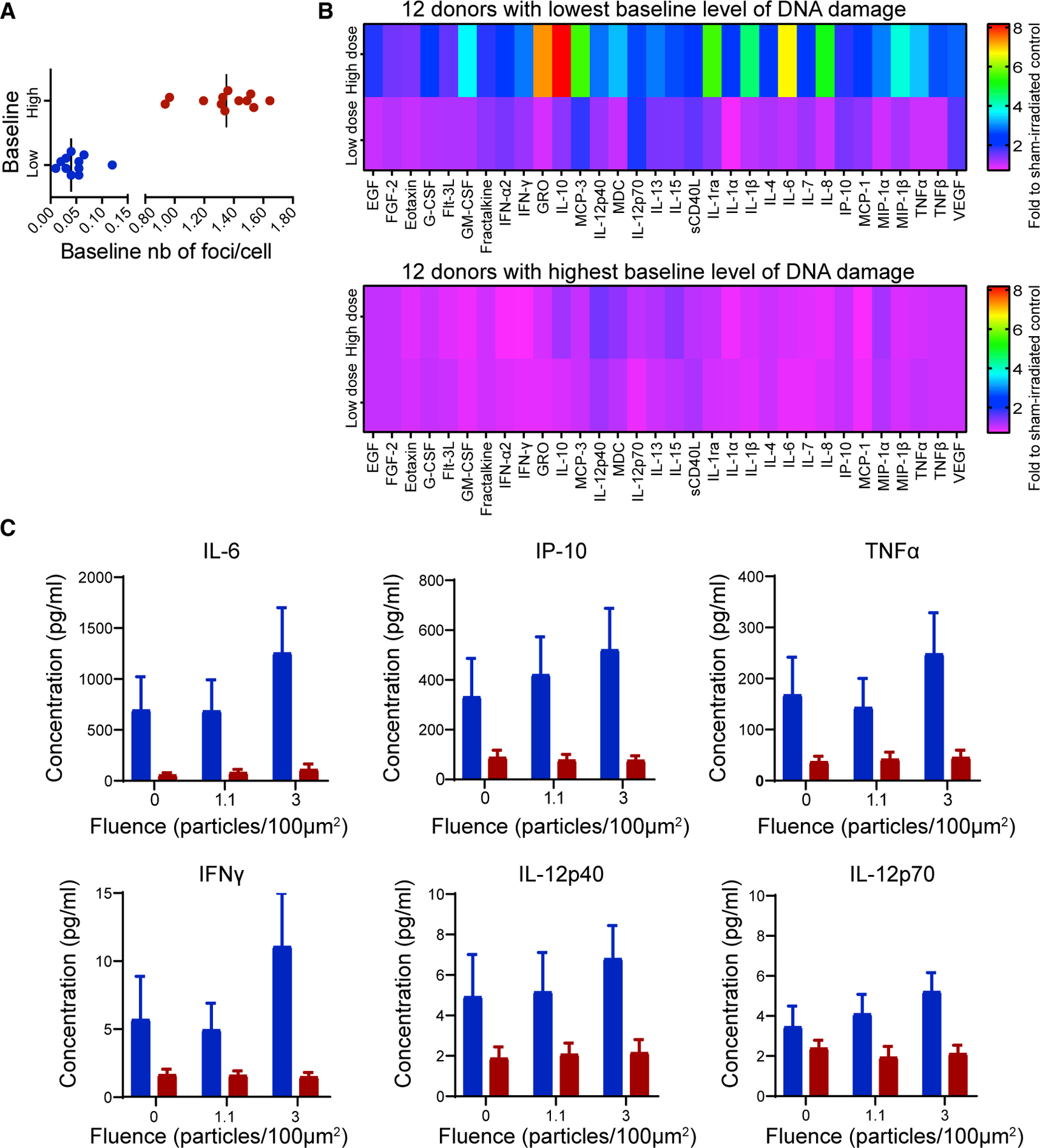
Higher Baseline DNA Damage Correlates with Lower Expression of Immune Cytokines (A) Comparison of baseline number of foci per cell in PBMCs from the 12 low baselines and the 12 high baselines of selected donors. (B) Heatmap of concentration of 32 immune cytokines relative to sham-irradiated control levels in PBMC supernatant collected at 24 h following 1.1 and 3 particles/100 µm^2 56^Fe irradiation for the 12 low and the 12 high baseline individuals. (C) Concentration for 6 of the 32 cytokines reported in (B) for the 12 low baseline (blue) and the 12 high baseline (red) individuals listed in (A). IL-6, **p = 0.0093; IP-10, p = 0.18; TNF-α, *p = 0.030; IFN-γ, *p = 0.042; IL-12p40, p = 0.34; IL-12p70, ***p = 0.0007; interaction p values, two-way ANOVA. Error bars, mean ± standard error. See also Figure S2.

Of all HZE components of GCR, we focused on the most carcinogenic particle, 600 MeV/n ^56^Fe (Alpen et al., 1993) for which the highest levels of DNA damage and the highest reduction in oxidative stress were observed and were related to the highest predicted amount of H_2_O_2_ production, as described earlier. Thus, the intracellular ^56^Fe response may be reflected in the molecules secreted in the supernatant of these cultures, specifically immunoregulatory cytokines. Exposure to ionizing radiation has indeed been shown to disrupt the immune system by altering the levels of inflammatory cytokines produced by PBMCs (Fernandez-Gonzalo et al., 2017). Thus, we used multiplex Luminex assay to quantify a panel of 32 standard human immune cytokines secreted for the first 24 h after exposure to 1.1 and 3 particles/100 µm^2^ of ^56^Fe irradiation in PBMCs from healthy donors who had extremely low or high levels of baseline DNA damage (Figure 7A, N = 12 donors per group).

A distinct response was observed in these two groups. No significant changes in cytokine expression were observed with fluence for the 12 donors with high levels of baseline DNA damage, and expression levels were close to control values (Figure 7B). However, the radiation response was radically different for the 12 donors with low levels of baseline DNA damage: most cytokines were expressed at levels higher than control after 1.1 particles/100 µm^2 56^Fe irradiation, and all cytokine expression increased significantly after 3 particles/100 µm^2^ (Figure S2). In addition, the baseline level of some cytokines, especially those associated with senescence (interleukin [IL]-6 and interferongamma-induced protein [IP]-10/C-X-C motif chemokine ligand 10) and with type I immune responses (tumor necrosis factor alpha [TNF-α], interferon [IFN] γ, IL-1α, and IL-12) (Glück et al., 2017; Mackenzie et al., 2017), increased even without irradiation in subjects with low compared with high baseline DNA repair and was exacerbated by irradiation in a dose-dependent manner (Figure 7C). This result might be caused by a different immune cell distribution, skewing toward Th1 and from Th2 in the low baseline DNA repair subjects, or a systemic environment that is sensitized to respond to perturbations. Thus, lower levels of baseline DNA damage correlate not only with higher levels of radiation-induced DNA repair but also with enhanced cytokine signaling after irradiation and at baseline for some cytokines relevant to the DNA damage response.

## DISCUSSION

In this study, we report that baseline levels of DNA damage foci in circulating peripheral blood lymphocytes isolated from venous blood draw or a finger prick are a powerful predictor of human immune responses to ionizing radiation and potential long-term health effects. Our study on simulated GCR showed that low numbers of baseline DNA damage foci are correlated with a higher RIF and cytokine response to the dose. Our study of cancer patients showed a correlation between low levels of baseline DNA damage and less severe clinical secondary effects after radiotherapy, whereas the opposite was true for individuals with high levels of baseline DNA damage foci. These trends underscore the potential for baseline DNA damage in lymphocytes to be a predictive biomarker of human responses to ionizing radiation in the context of space biology, as well as cancer radiotherapy. We have shown similar correlations between both levels of baseline DNA damage and persistent radiation-induced DNA damage in bronchial cells with susceptibility to radiation-associated lung cancer in 17 strains of mice (Ochola et al., 2019).

In healthy humans, DNA damage can occur spontaneously, or it can be induced by environmental stressors. Our reported positive correlation between age and DNA damage has also been identified previously and is mainly attributed to impaired DSB repair with increasing age (Frasca et al., 1999; Garm et al., 2013; Løhr et al., 2015; Rübe et al., 2011). We were able to confirm the age dependence using a single marker of baseline 53BP1 foci number per cell, which indicates that this repair protein encompasses enough of the DNA damage and repair response to properly characterize inter-individual DNA damage variability and suggests its potential adaptation for point-of-care biomarker systems.

This work also highlights factors relevant to experimental design that can influence baseline DNA damage foci levels: (1) PBMC-subtype composition, (2) sample processing, and (3) blood microenvironment. First, we reported significantly lower levels of baseline foci in healthy donor T lymphocytes isolated from a finger prick compared with PBMCs isolated from blood draw. However, despite the differences in sample collection and isolation, the two methods showed similar trends for predicting DNA damage responses, which illustrates the robustness of baseline foci levels as a predictive biomarker.

Sham-irradiated controls that were frozen and plated in new media showed even higher foci levels, indicating that the freezing process further induces DNA damage. Interestingly, samples that were removed from their original microenvironment also had the lowest variance among individuals, suggesting that at least part of the individual variation at baseline is driven by the blood microenvironment. The same blood microenvironment, which is affected by lifestyle factors such as nutrition, fitness, chronic health conditions, and the gut microbiome, could modulate the therapeutic radiation response, contributing to further inter-individual variabilities. While interpreting our results, we kept in mind the additional role that the blood microenvironment played in the cancer patient samples, whereas the simulated cosmic radiation experiment using *ex vivo* irradiations of frozen and recultured PBMCs is expected to be primarily driven by genetic and epigenetic differences among donors.

PBMC-subtype distribution has been studied as a predictor of clinical outcomes to radiotherapy (Braun et al., 2019; Grassberger et al., 2019; Saber et al., 2016). Specifically, the ratio of CD4^+^/CD8^+^ T lymphocytes has been shown to correlate with radiotherapy outcomes (An et al., 2019; Hoppe et al., 1995; Lee et al., 2009). Another study of *ex vivo* radiation-induced responses in both CD4^+^ and CD8^+^ T lymphocytes was able to predict late radiotherapy effects (Ozsahin et al., 2005). In future studies, it would be interesting to evaluate the predictive potential of combining these two biomarkers—PBMC profiling and baseline DNA damage foci analysis—to establish an even more sensitive predictor for immune responses to ionizing radiation exposure.

The DNA damage quantification method proposed in this paper is based on immunocytochemistry of DNA repair protein 53BP1. Reduction in 53BP1 foci can be driven by two opposite mechanisms depending on the context: less DNA damage or disruption of the DNA repair pathway (poor recruitment of 53BP1). Specifically, when studying baseline levels of 53BP1 foci, we lack information about the kinetics of 53BP1 recruitment, because we do not know when the damage occurred. Therefore, the quantity of 53BP1 foci in baseline samples reflects a steady-state equilibrium between spontaneous damage and continuous repair (Tang et al., 2007). Spontaneous damage can be reduced by the presence of antioxidants in the blood and other potential biomolecules found circulating in healthy individuals. However, when inducing DNA damage using a known stressor such as ionizing radiation, we take the system off the steady state, and 53BP1 foci quantification primarily provides information on the repair kinetics and efficiency of 53BP1 recruitment. However, even in the context of radiation exposure, a healthy microenvironment may help reduce the amount of damage or even improve the repair machinery. In this context, higher RIF response is correlated with lower levels of baseline foci, suggesting that low baseline individuals have an efficient repair process with strong repair protein recruitment and such efficiency may be driven by both genetics and microenvironmental factors.

To validate this hypothesis, it will be important to identify whether low baseline individuals present a particular genetic background, in particular in areas involved in the DNA damage response. To that end, we genotyped all 674 healthy donors after collecting PBMCs for associations to radiation responses. Although this study is ongoing, preliminary results in mice (Pariset et al., 2020) indicate associations between the SNPs located in genes involved in DNA repair pathways and the kinetics and efficiency of repair for 48 h after exposure to gamma or high-LET (350 MeV/n ^40^Ar and 600 MeV/n ^56^Fe) radiation.

The correlation between baseline levels of 53BP1 foci and clinical secondary effects after radiotherapy demonstrates that baseline levels of DNA damage can be used to predict long-term effects. Adjusting the radiation dose and fractionation schedule can modulate the peripheral immune response after radiotherapy (Zhang et al., 2017). Increased fractionation is important for reducing normal tissue toxicity (Turesson and Notter, 1984), but it leads to poorer local tumor control (Maciejewski et al., 1990). Results from our study indicate that individuals who have low levels of baseline 53BP1^+^ foci can be considered a resilient population that has reduced toxicity in normal tissues after radiotherapy, likely due to more efficient DNA repair machinery. These individuals would benefit from more aggressive treatment via hypofractionation and flash therapy, which would improve tumor control and significantly reduce the cost of treatment. However, the mechanisms of radiation-induced acute effects during radiation therapy or radiological accidents are triggered and modulated by other biological and physiological pathways. DNA damage and repair processes are important mechanisms, but not the only mechanisms, responsible for these events (Citrin and Mitchell, 2017).

We observed that low levels of baseline DNA damage were associated not only with higher levels of 53BP1^+^ foci after irradiation, presumably because of better recruitment of DNA damage repair proteins, but also with significantly more changes in immune cytokine expression. In contrast with intranuclear responses reflected in DNA damage and repair patterns, cytokine expression patterns represent global tissue responses to irradiation, mediated by oxidative stress, cellular senescence, impaired cellular functions, and cell death. Pro-inflammatory cytokines are required for normal detection and clearance of radiation-damaged cells, and anti-inflammatory cytokines prevent the immune response from persisting and spreading into healthy tissue. Thus, the normal dose-response behavior for induction of both pro- and anti-inflammatory cytokines in cells from individuals who have low baseline levels of DNA damage is likely associated with not only a more efficient DNA repair response but also a better cellular repair response (Åhsberg et al., 2010), promoting the clearance of cells that are either killed or damaged by radiation and may become dysfunctional or cancerous.

Radiation-dependent changes in cytokine and chemokine expression suggest an increase in hematopoiesis and immune differentiation (Flt-3L, fibroblast growth factor 2 [FGF-2], and epidermal growth factor [EGF]) (Åhsberg et al., 2010; Doan et al., 2013; Itkin et al., 2012), which has previously been reported in radiation-induced injury (Doan et al., 2013), as well as adhesion and migration of differentiated immune cells (e.g., fractalkine) (Tang et al., 2007). Other significantly affected cytokines regulate inflammation, combining immunity mediated by pro-inflammatory Th1 lymphocyte (IL-12p70, Wagner et al., 2004, and IL-15, Santana Carrero et al., 2019) and Th2 lymphocyte (eotaxin and IL-13; Li et al., 1999) with anti-inflammatory responses (IFN-a2; Paul et al., 2015), as well as increased angiogenesis (VEGF and FEGF-2; Ferrara, 2002; Smith et al., 2013). Altogether, these changes in cytokine and chemokine expression could contribute to a positive response to radiotherapy via immune cell recruitment into the tumor, consistent with our results that patients with low levels of baseline DNA damage have fewer side effects after radiotherapy treatment. However, the same response may be either helpful or harmful after exposure to space radiation: although increased inflammation would help clear dead cells, it may exacerbate immune dysregulation that has been observed in astronauts in low-Earth orbit (Crucian et al., 2008, 2015).

In summary, our results indicate that baseline DNA damage in human blood immune cells, quantified based on 53BP1^+^ foci number, is inversely associated with levels of *in vivo* and *ex vivo* ionizing RIF levels. This suggests a more effective recruitment of DNA damage repair mechanisms in individuals with low levels baseline DNA damage, which was shown to be beneficial for cancer patients by reducing the secondary effects to radiotherapy and might be beneficial for astronauts by limiting the damage caused by exposure to deep space radiation. However, the potential utilization of 53BP1+ baseline expression as a biomarker for space radiation sensitivity would require further analysis, especially in conditions of whole-body *in vivo* irradiation, which was not covered in our experiments. In addition, further experiments would be useful in teasing out the impact of circulating factors in blood and especially free radical scavengers, which can serve as potential antioxidants and DNA repair enhancers, by studying the plasma of low and high baseline individuals with DNA damage. Ideally, results would also be replicable in the full spaceflight environment that combined a low dose rate of GCR irradiation with simultaneous exposure to other stressors, including microgravity, high carbon dioxide concentration, and physiological and social stress caused by social isolation and circadian rhythm disruption.

## STAR★METHODS

### RESOURCE AVAILABILITY

#### Lead contact

Further information and requests for resources and reagents should be directed to and will be fulfilled by the Lead Contact, Sylvain V. Costes (sylvain.v.costes@nasa.gov).

#### Materials availability

This study did not generate new unique reagents.

#### Data and code availability

This study did not generate any unique datasets or code.

### EXPERIMENTAL MODEL AND SUBJECT DETAILS

#### Cohorts of healthy donors

Buffy coat samples were collected from 674 donors of European descent, 47% males, 53% females, 18–75 years old. The following exclusion criteria were used to select healthy donors: history of neurological disorders, history of or current autoimmune disease, cancer, and infections. Among this population, 319 donors were randomly selected (first in order of collection) for *in vitro* radiation-induced DNA damage and oxidative stress measurements presented in this manuscript. Of these 319 donors, 24 individuals were selected based on their extreme baseline level of DNA damage: 12 ‘‘low baseline’’ donors, 58% females, 42% males, 19–68 years old, and 12 ‘‘high baseline’’ donors, 67% females, 33% males, 20–64 years old. All procedures were approved by the Institutional Review Board at NASA Ames Research Center (IRB# HRI-357 and HRI-358) and complied with all ethical principles and guidelines for research involving human subjects. BMI groups were defined based on classification from the Centers for Disease Control and Prevention (CDC): underweight for BMI below 18.5, normal for BMI between 18.5 and 24.9, overweight for the range 25.0 – 29.9 and obese above 30.0.

Finger prick samples were collected from 339 donors, 20–74 years old, who were asked if they had any sickness, recent medical imaging, infection, or ongoing cancer at the time of collection. Individuals with any of these recent potential confounding factors were removed from this study. This study was approved by Ethical & Independent Review Services (E&I) (5710 Paradise Drive, Suite 11, Corte Madera, CA, 94925., E&I West Coast Board, Chair of the Board, Jean Taylor-Woodbury), roster dated January 1, 2015, IRB #00007807 valid from 12/29/2015 to 12/28/2016 (E&I PI#: 16903-001, Study #: 15123-01). Individuals (four donors) who previously had cancers have been kept and labeled in Figure 3.

#### Cohort of prostate cancer patients

Patients diagnosed with prostate cancer without prior history of cancer or radiotherapy (RT) were recruited while undergoing prostate proton radiotherapy at the Loma Linda University (LLU) James M. Slater, MD. Proton Treatment and Research Center (PTRC), Loma Linda, CA, USA. All procedures were approved by the Institutional Review Board at Loma Linda University (IRB# 5140189) and complied with all ethical principles and guidelines for research involving human subjects. This cohort comprised 37 male patients with a median age of 70 ± 7.2 years. 30 patients were included in this paper, based on data availability on secondary effects of treatment.

### METHOD DETAILS

#### MCF10A cell culture and immunostaining

Nonmalignant human mammary epithelial cells (MCF10A, ATCC) were cultivated in minimum essential medium (MEM, Invitrogen), supplemented with bovine pituitatry hormone (13 mg/mL), hydrocortisone (0.5 mg/mL), hEGF (10 µg/mL), insulin (5 mg/mL), and cholera toxin (100 ng/mL) (Invitrogen). Cell culture was performed in Permanox plastic 8-well Lab-Tek chamber slides (Nalge Nunc International Corporation) at 37C, with 95% humidity and 5% CO_2_. The cells were grown to a confluent layer prior to irradiation. After three washing steps with PBS, cells were blocked with 0.2% BSA for 15 min, incubated with the primary antibodies for 15 min, washed with PBS, and incubated with the secondary antibodies for 15 min. The primary antibodies were a rabbit polyclonal anti 53BP1 antibody (1:100 from 1 mg/mL, Bethyl Laboratories) and a mouse monoclonal to phosphohistone H2AX antibody (1:100 from 1 mg/mL, clone JBW301, Upstate Cell Signaling Solutions Inc.). The corresponding secondary antibodies were either FITC-labeled antirabbit IgGs, or T-Red-labeled antimouse IgGs (Molecular Probes Invitrogen). After a further washing step with PBS, the cells were counterstained with DAPI. All steps were performed at room temperature.

#### Conformal proton treatment

Patients were treated with protons at the PTRC 70–250 MeV synchrotron. Proton radiotherapy was delivered by means of opposed lateral beams; in most cases one field is treated each day with incident proton energies ranging from 225–250 MeV. The planned target volume included the prostate, seminal vesicles with 5-mm margin for setup uncertainty. Treatment cohorts were divided in low- and high-risk-patients based on staging (T1a to T3a), prostate specific antigen (PSA) levels, and Gleason index (GI). For low risk patients with of pelvic lymph node involvement were treated to 81 Gy at 1.8 Gy per fraction to the prostate and seminal vesicles with proton therapy alone. For high risk patients, the standard prescribed dose is 36 Gy at 1.8 Gy per fraction/day. The proton treatment is followed by pelvic photon irradiation of 45 Gy at 1.8 Gy/fraction (Slater et al., 2004). The integral dose was calculated by contouring the treatment regions and the patients’ bodies for the treated areas for each patient’s CT scan treatment plan. Dosages to the seminal vesicular areas were estimated by a computer model using the beam characteristics used for the patient.

#### Clinical evaluation

To assess acute toxicity, each patient was asked to complete the Expanded Prostate Cancer Index Composite survey (EPIC) and the treating radiation oncologist filled a consolidated prostate cancer clinician data collection matrix based on the Common Terminology Criteria for Adverse Events (CTCAE) Version 4.0 (Basch et al., 2014). These clinical surveys allowed measuring gastro intestinal system (GI), renal/urinary system (RU), skin, and fatigue, vascular, reproductive, and psychological health. Both EPIC and the consolidate matrix collection data were employed before, mid-treatment, and at the end of treatment. Individual patient scores for analyses were determined as the number grade 2 or 3 of acute toxicities that began or increased since the start of treatment, with pre-treatment serving as baseline. Patients were sorted into ‘‘non-reactive’’ (zero incidence of acute effects), ‘‘normally reactive’’ (1 to 4 acute toxicity events) and ‘‘overly reactive’’ (more than 4 acute toxicity events) at the time of the treatment completion (2 months).

#### Blood sampling from cancer patients

Prior to the first proton treatment fraction, 10mL of each patient’s blood was drawn and collected into heparinized tubes. Blood samples were collected by adding 50 µL of blood to sample tubes pre-coated with 45 µL of 2% paraformaldehyde (PFA) and 5 µL of 0.5M EDTA, and the tube was incubated at room temperature for at least 1 h to allow for complete fixation. Additional 1mL blood aliquots were irradiated with protons *in vitro*. An identical procedure was carried out using photons from a Co^60^ gamma source. Immediately after irradiation, 50 µl of irradiated blood was incubated in RPMI medium. At 24 hr after irradiation, RPMI-blood mixture samples were fixed as described above. At the proton treatment mid-point (1 month), blood was drawn and 100 µl aliquots were fixed as described previously to assess the *in vivo* foci formation. Blood draw was performed on Tuesdays or Thursdays to avoid repair effects that potentially occurred during the weekend.

#### Photon and proton *in vitro* and *in vivo* irradiation

Blood irradiation with photons and protons was completed at the PTRC, Loma Linda California. For these irradiations, 250 MeV therapeutic proton beams were modulated to generate a 5.0 cm wide spreadout Bragg peak (SOBP). The blood samples were located at a water equivalent depth of 29.6 cm, specified using CIRS plastic water blocks, which placed the cells in the uniform dose SOBP region of the proton dose profile. Fresh blood samples were exposed to protons and gamma rays in 15ml centrifuge tubes in a dedicated acrylic sample holder. Protons were delivered in a pulsed fashion, with a pulse duration of 0.125 s and a duty cycle of 2.2 s. This pulsed modality of beam delivery gave a dose rate of approximately 0.8 Gy/min and blood samples were exposed to single dose of 0, 0.5, 1 or 4 Gy. Photon cell irradiations were done using a Co^60^ irradiator (Eldorado Model ‘G’ machine, Atomic Energy of Canada Ltd., Commercial Products Division, Ottawa, Canada) with a dose rate of 1Gy/min at 80cm source to sample distance (SSD) and field size of 40x40 cm^2^.

#### Blood Sampling from Healthy Donors

Buffy coat samples were purchased from Oklahoma Blood Institute within 24 h of collection from consented donors (NASA HRP #NNJ16HP24I, NASA Ames Research Center Human Research IRB #HRI-357). All blood samples were tested for Zika virus, atypical antibodies, Chagas disease, Hepatitis B, Hepatitis C, Human Immunodeficiency Virus, Human T-lymphotropic Virus, syphilis, and West Nile Virus. 156 donors were randomly selected to be tested for Cytomegalovirus.

Finger prick blood samples were collected by adding 50 µL of blood to sample tubes pre-coated with 45 µL of 2% paraformaldehyde (PFA) and 5 µL of 0.5M EDTA, and the tube was incubated at room temperature for at least 1 h to allow for complete fixation. Before lymphocyte isolation, the blood and fixative mixture (about 100 µL) was resuspended by pipetting up and down 30–50 times and transferring to a 96 well Ibidi plate (Ibidi LLC., Cat #89626).

#### PBMC isolation

Buffy coat samples were diluted 1:1 with PBS, and PBMCs were isolated from the diluted buffy coat by layering 38 mL buffy coat onto 12 mL Ficoll-Paque (VWR Cat# 95038-168) and centrifuging at 300 x g for 25 min without brakes. The cloudy layer containing PBMCs was collected, washed with PBS, incubated with red blood cell lysis buffer (VWR Cat# 420301) for 5 min at room temperature, and finally washed again with PBS. Cells that were being evaluated for baseline DNA damage were transferred to a 96-well u-bottom plate, 3x10^6^ cells per well, and fixed with 2% PFA for 15 min and then stored at 4°C until immunostaining. Cells that were being stored for future irradiations were suspended at 10^7^cells/mL in 10% DMSO in FBS and kept in a Mr. Frosty freezing container (ThermoFisher Cat# 5100-0001) at −80°C for a few days for gradual cooling before being transferred to −150°C for long term storage.

CD3+ lymphocytes were isolated from fixed blood samples - collected via finger prick for healthy donors and blood draw for cancer patients - using the EasySep Human Whole Blood CD3 Positive Selection Kit (StemCell Tech Cat# 18081). Briefly, fixed blood was incubated with 100 µL of red blood cell lysis buffer (StemCell Tech Cat# 20110) for 15 min at room temperature in the 96-well plate. After incubation, 4.5 µL of the CD3 Positive Selection Cocktail (StemCell Tech Cat# 18081C.1) was added to each well and incubated for 15 min at room temperature. Next, 4.5 µl of the Magnetic Nanoparticles Positive Selection reagent (StemCell Tech Cat# 18150) was mixed into each well and incubated for 15 min at room temperature. All wells were washed with Washing Medium (2% FBS 1 mM EDTA in PBS). The plates were then incubated on a magnet at room temperature for 15 min, then the supernatant was removed and beads were rinsed thoroughly using Washing Medium. 10 min magnet incubations and washes were repeated 5–20 times until no red color was detected (to ensure maximum removal of red blood cells). The cells were finally resuspended in Washing Medium and transferred to a new 96-well Ibidi plate pre-coated with Cell-tak (BD Cat# 2173828) for immunostaining. The Cell-Tak coated plates were prepared by mixing 47.5 µL of 0.1 M sodium bicarbonate with 2.5 µL of Cell-Tak and incubating the solution in the plate for 20 min at room temperature followed by washing with distilled water and an overnight incubation at 4°C.

#### GCR-like *in vitro* irradiation

PBMCs from buffy coat samples were thawed and plated in fibronectin coated 96-well Ibidi plates (Ibidi LLC Cat# 89626) and 96-well u-bottom plates (Corning Cat# 351177) 24 h before irradiation in RPMI 1640 (Sigma Cat# R8758) supplemented with 10% fetal bovine serum (VWR Cat# 1300-500H) and 1% Penicillin/Streptomycin/Glutamine (Fisher Cat# 10-378-016). Each well received approximately 1.6x10^6^ cells and 2mL media. 24 h later, the plates were irradiated with 0, 1.1, or 3 particles/100µm^2^ of 350 Mev/n ^28^Si, 350 MeV/n ^40^Ar, or 600 MeV/n ^56^Fe or 0Gy, 0.1Gy or 1Gy of gamma rays. MCF10A cells were irradiated with 1 Gy of 1 GeV/n ^56^Fe ions, delivered at a dose rate of 100 cGy/min. Irradiations were carried out at NASA Space Radiation Laboratory (NSRL) at Brookhaven National Laboratory (BNL). Plates were irradiated with the beam hitting below the plate, and the plate angled at less than 5 degrees. Dose rate for both gamma rays and ion irradiations was 1 Gy/min. At 4 h post irradiation, supernatant from the Ibidi plates was removed and frozen at −80°C until cytokine quantification, and the cells were fixed with 2% PFA for 15 min and stored at 4°C until immunostaining. The cells in the u-bottom plates were immediately stained for flow cytometry analysis.

#### Immunostaining

Fixed irradiated PBMCs isolated from buffy coat samples were permeabilized with 0.1% Triton X (Sigma Cat# T8787) in PBS for 20 min and then washed. All washes were performed with 0.1% Tween 20 (Sigma Cat# P1379) in PBS. Cells were blocked with 3% bovine serum albumin (Sigma Cat# A9647) in PBS for 1 h at room temperature before adding a 1:400 dilution of rabbit anti-53BP1 (Bethyl Labs Cat# IHC-00001) in blocking solution. After 1 h, the cells were washed and a 1:400 dilution of Alexa Fluor 488 goat anti-rabbit (ThermoFisher Cat# A11034) in blocking solution was added. After 1 h in the dark, cells were washed and a 1:1000 dilution of DAPI (ThermoFisher Cat# 62248) in PBS was added for 10 min. A final wash was performed and the cells were stored at 4°C protected from light until imaging.

Lymphocytes on Cell-Tak coated plates were incubated on a magnet for 5 min at room temperature and the wash solution was removed. The cells were permeabilized by a 5 min −20°C incubation with 100% ice-cold methanol. The plates were then placed on the magnet for 10 min at room temperature and the methanol was removed. The plates were washed with PBS and incubated at room temperature for 1 h with the same blocking solution mentioned above. Blocking solution was removed following a 10 min room temperature incubation on the magnet, and a 1:500 dilution of rabbit anti-53BP1 (Bethyl Labs Cat# A300-272A) in blocking solution was added to each well and incubated at 37°C. After 1 h, the plates were incubated on the magnet for 10 min at room temperature, the primary antibody solution was removed, and the wells were washed with PBS. A 1:400 dilution of Alexa 488 goat anti-rabbit (ThermoFisher Cat# A11034) in blocking solution was added to the wells and incubated in the dark at 37°C for 1 h. Plates were placed on the magnet again for 10 min at room temperature, the secondary antibody solution was removed, and the wells were washed with PBS. A 1:1000 dilution of DAPI (ThermoFisher Cat# D1306) in PBS was added to each well and incubated for 5 min in the dark at room temperature on the magnet. Finally, the DAPI solution was removed, the plates were washed with PBS, and 2 drops of Mounting Media (Trevigen Cat# 4866-20) were added to each well. Plates were stored at 4°C protected from light until imaging.

#### Imaging

Fixed and stained cells were imaged using two semi-automated setups. The first setup involved an Axiovert epifluorescence microscope (ZEISS) with a plan-apochromat 40X NA0.95 dry objective (ZEISS), coupled to a continuous reflection interface sampling and positioning system (CRISP, ASI) controlled by MATLAB (Mathworks). Briefly, the wells were scanned to find cells based on DAPI detection, and only regions with optimal cell density (between 2 and 100 nuclei per field of view) were selected for foci imaging. For each selected field, one image slice was taken for DAPI and a z stack of 9 image slices spaced 1 µm apart were taken for FITC. Image segmentation and detection was performed on the fly in MATLAB (Mathworks). Once 800 cells were imaged in one well, the camera moved to the next well. If by 40 positions the 800 cell target was not reached, the camera moved on to the next well.

The second imaging setup used the CellDiscoverer 7 (CD7) Microscope (ZEISS) controlled by the CD7 Zen imaging software. Briefly, 15 positions evenly spaced throughout each well were imaged using preset settings for DAPI and AF488 detection. A single image was taken for DAPI images, and 21 image slices were taken for AF488, spaced 0.49 µm apart. Images were acquired at 20X magnification.

Raw images obtained from both imaging setups were analyzed using MATLAB (Mathworks) and the image processing toolbox for MATLAB, DIPImage (Delft University of Technology, the Netherlands). A mask for each nucleus was created based on the DAPI image, and the number of FITC foci within each mask was recorded, using a pattern recognition approach by applying a wavelet morphological filter combined with a watershed algorithm to separate touching RIF. The same threshold of FITC fluorescence was applied to all images for identifying foci.

#### Oxidative stress quantification

Oxidative stress was quantified via flow cytometry in the plated PBMCs isolated from buffy coat samples at 4 h after irradiation. Staining was performed in the u-bottom plates using the CellROX Green Flow Cytometry Assay Kit (ThermoFisher Cat# C10492) per the manufacturer’s instructions. Briefly, the CellROX reagent was diluted in DMSO and PBMC media and then added to live cells and incubated for 45 min at 37°C. Cells were then fixed with 2% PFA for 15 min at 4°C protected from light. Finally, the cells were washed with PBS and transferred to flow cytometry tubes. Flow cytometry was performed using a BD FACSCalibur flow cytometer and analyzed using FlowJo™ Software (Ashland, OR: Becton, Dickinson and Company; 2019).

#### Cytokine quantification

PBMC supernatants from selected subjects (12 ‘‘high baselines’’ and 12 ‘‘low baselines’’) were analyzed for cytokine content 24 h following 1.1 or 3 particles/100 µm^2 56^Fe irradiation. The supernatants were thawed at 4°C and 25 µL from each sample was used with the MILLIPLEX MAP Human Cytokine/Chemokine Magnetic Bead Panel - Immunology Multiplex Assay (Millipore Cat# HCYTOMAG-60K) per the manufacturer’s instructions. This kit allowed for the simultaneous quantification of 41 analytes: EGF, Eotaxin, G-CSF, GM-CSF, IFNα2, IFNγ, IL-10, IL-12P40, IL-12P70, IL-13, IL-15, IL-17A, IL-1RA, IL-1α, IL-1β, IL-2, IL-3, IL-4, IL-5, IL-6, IL-7, IL-8, IP-10, MCP-1, MIP-1αa, MIP-1β, RANTES, TNFα, TNFβ, VEGF, FGF-2, TGF-α, FIT-3L, Fractalkine, GRO, MCP-3, MDC, PDGF-AA, PDGF-AB/BB, sCD40L, and IL-9. Briefly, a 96-well flat bottom sample plate was washed for 10 min on a shaker at room temperature using the Wash Buffer (Cat# L-WB). After removing the Wash Buffer, the standard and control wells received 25 µL of each standard (Cat# MXH8060) dilution or control (Cat# MXH6060) and 25 µL of PBMC culture medium, while sample wells received 25 µL of the Assay Buffer (Cat# L-AB) and 25 µL of the appropriate sample. Finally, 25 µL of sonicated and vortexed premixed beads (one bead solution for all 41 analytes) (Cat# HCYPMX38-MAG, HCYRNTS-MAG, HPDGFAA-MAG, HPDGFBB-MAG) was added to all wells and incubated in the dark on a shaker overnight at 4°C. After the incubation was complete, the wells were emptied and washed twice with the Wash Buffer using a magnet (EMD Millipore Cat# 40-285). Each well received 25 µL of room temperature Detection Antibodies (Cat# MXH1060-4) followed by a 1 h incubation on a plate shaker at room temperature protected from light. Then, 25 µL of Streptavidin-Phycoerythrin (Cat# L-SAPE11) was added to each well and incubated again on a shaker in the dark at room temperature for 30 min. Next, the wells were emptied and washed twice with the Wash Buffer using a magnet. Finally, 150 µL of Drive Fluid (EMD Millipore Catalog # MPXDF-4PK) was added to each well, the plate was agitated on a shaker for 5 min to resuspend the beads, and the plate was read in a MAGPIX® with xPONENT® software. The median fluorescent intensity (MFI) data were saved and analyzed using a 5-parameter logistic method to calculate the concentration of each analyte per sample. Here we only report the results for 32 of the analytes, because the concentrations for IL-17A, IL-2, IL-3, IL-5, RANTES, TGF-α, PDGF-AA, PDGF-AB/BB, and IL-9 were below the detection limit for some samples.

#### RITRACKS calculations

Radiation chemistry calculations were done with the software RITRACKS. This software comprises several interrelated codes, written in C++, that are used for different purposes such as radiation track structure, radiation chemistry, micro- and nano-dosimetry, and DNA damage. The main code simulates the detailed stochastic radiation track structures from various types of ionizing radiations, especially heavy ions, electrons, and photons. The code simulates all ionizations and excitations of the incident radiation in the medium, and follows those resulting from the interactions of the ionized electrons, which generates the track structures (Plante and Cucinotta, 2011). The radiation chemistry part is based on the Green’s functions of the diffusion equation, and the calculations can be made either by the step-by-step (SBS) or independent reaction times (IRT) methods (Plante and Devroye, 2017). For the simulations done for this work, the SBS and IRT methods yields almost identical results.

### QUANTIFICATION AND STATISTICAL ANALYSIS

Only samples with more than 50 nuclei for DNA damage (based on high-throughput imaging) and more than 200 cells for oxidative stress (based on flow cytometry forward and side scatter signals) were kept for the analysis. Batch effects were corrected using Com-Bat (Johnson et al., 2007) from the surrogate variable analysis package of R (Leek et al., 2012). Statistical analyses were performed using Prism and stats R package for ANOVA, Tukey’s post hoc tests and t tests (R Core Team, 2013). The level of significance used in this study was p = 0.05.

## Supplementary Material

1

2

## Figures and Tables

**Table:** KEY RESOURCES TABLE

REAGENT or RESOURCE	SOURCE	IDENTIFIER
Antibodies		
Rabbit anti-53BP1	Bethyl Labs	Cat# IHC-00001; RRID: AB_513644
Alexa Fluor 488 goat anti-rabbit	ThermoFisher	Cat# A11034; RRID: AB_2576217
DAPI	ThermoFisher	Cat# 62248
Rabbit anti-53BP1	Bethyl Labs	Cat# A300-272A; RRID: AB_185520
DAPI	ThermoFisher	Cat# D1306
Mouse monoclonal anti-γH2AX antibody	Upstate Cell Signaling Solutions	Clone JBW301; RRID: AB_310795
TexasRed-labeled anti-mouse	Molecular Probes Invitrogen	Cat# T-862; RRID: AB_2556781

Biological Samples		

PBMCs from buffy coat	Oklahoma Blood Institute	N/A
CD3+ cells from finger prick collection blood	Exogen	N/A
CD3+ cells from total blood draw collection	Loma Linda University	N/A
MCF10A cells	ATCC	CRL-10317

Chemicals, Peptides, and Recombinant Proteins		

Ficoll-Paque	VWR	Cat# 95038-168
Red blood cell lysis buffer	VWR	Cat# 420301
Triton X-100	Sigma	Cat# T8787
Tween 20	Sigma	Cat# P1379
Bovine serum albumin	Sigma	Cat# A9647
Cell-Tak	BD	Cat# 2173828
Mounting Media	Trevigen	Cat# 4866-20

Critical Commercial Assays		

EasySep CD3+ Whole Blood Selection Kit	StemCell Technologies	Cat# 18081
CellROX Green Flow Cytometry Assay Kit	ThermoFisher	Cat# C10492
MILLIPLEX MAP Human Cytokine/Chemokine Magnetic Bead Panel - Immunology Multiplex Assay	Millipore	Cat# HCYTOMAG-60K

Software and Algorithms		

MATLAB	MathWorks	https://www.mathworks.com/products/matlab.html
DIPImage	Delft University of Technology	http://www.diplib.org/download
FlowJo™ Software	BD	https://www.flowjo.com/

Other		

CellDiscoverer 7	ZEISS	SR# 4649000100
CRISP Autofocus System	ASI	PR# TG-1000-8
Plan-apochromat 40X NA0.95 dry objective	Zeiss	Cat# N010098
ORCA-Flash4.0 LT+ Digital CMOS camera	Hamamatsu	Cat# C11440-42U30
FACSCalibur flow cytometer	Becton Dickinson	Cat# 342973

## Highlights

- Baseline DNA damage in human immune cells correlates with age and latent infection
- Low baseline DNA damage results in better clinical outcomes after radiotherapy
- Low baseline DNA damage correlates with stronger DNA repair response to irradiation
- Low baseline DNA damage correlates with higher inflammatory cytokine dose response
