# Supplementary material for: DNA Damage Baseline Predicts Resilience to Space Radiation and Radiotherapy
Source: Cell Rep. Author manuscript; Available in PMC 2021 Jan 5. (PMC7784531; doi:10.1016/j.celrep.2020.108434)
Supplement: 1 [file NIHMS1653533-supplement-1.pdf]

Cell Reports, Volume *33*

Supplemental Information

DNA Damage Baseline Predicts Resilience to Space Radiation and Radiotherapy

Eloise Pariset, Antonella Bertucci, Margaux Petay, Sherina Malkani, Alejandra Lopez Macha, Ivan G. Paulino Lima, Vanesa Gomez Gonzalez, Antony S. Tin, Jonathan Tang, Ianik Plante, Egle Cekanaviciute, Marcelo Vazquez, and Sylvain V. Costes

Figure S1. Baseline level of spontaneous DNA damage in PBMCs increased with age, Related to Figure 2B and Figure 3A. Distribution of baseline number of foci/nucleus as a function of age in A. PBMCs extracted from blood samples from 674 healthy donors using a Ficoll-based protocol (** $p = 0.0093$, one-way ANOVA), and B. CD3+ lymphocytes extracted from finger prick samples from 339 healthy donors using a bead-based protocol (*** $p < 0.0001$, one-way ANOVA). Column bars showing mean ± standard error of the mean. Age groups defined as [18;25],]25;35],]35;45],]45;55],]55;65], and]65;75].

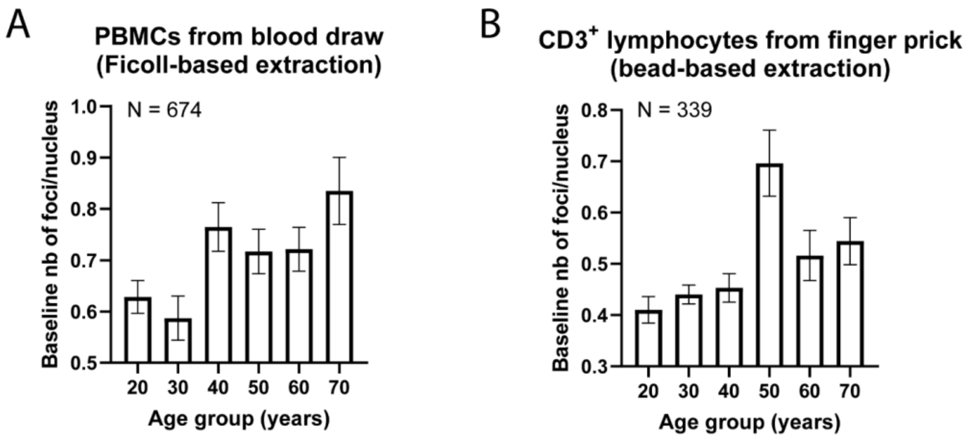

Figure S2. Higher baseline DNA damage correlates with lower expression of immune cytokines, Related to Figure 7. Concentration of the 26 cytokines reported in Figure 7B, and not shown in Figure 7C, for the 12 low baseline (black) and the 12 high baseline (grey) individuals listed in Figure 7A. IL-8: $p = 0.25$, IL-1RA: * $p = 0.017$, IL-10: p=0.18, IL-1β: ** p=0.0057, IL-1α: ** p=0.0053, IL-4: * p=0.016, IL-7: * $p = 0.039$, IL-13: ** $p = 0.0021$, IL-15: ** $p = 0.0059$, GRO: $p = 0.062$, MCP-1: $p = 0.41$, MCP-3: $p = 0.38$, MDC: $p = 0.15$, MIP-1β: $p = 0.75$, MIP-1α: $p = 0.81$, G-CSF: ** $p = 0.0024$, Fractalkine: *** $p = 0.0002$, VEGF: ** $p = 0.0027$, FGF-2: * $p = 0.014$, Flt-3L: *** $p = 0.0005$, GM-CSF: $p = 0.70$, Eotaxin: * $p = 0.013$, TNF-β: * $p = 0.041$, and **** $p < 0.0001$ for IFN-α2, sCD40L, EGF, interaction p-values, 2-way ANOVA. Error bars, mean ± standard error.

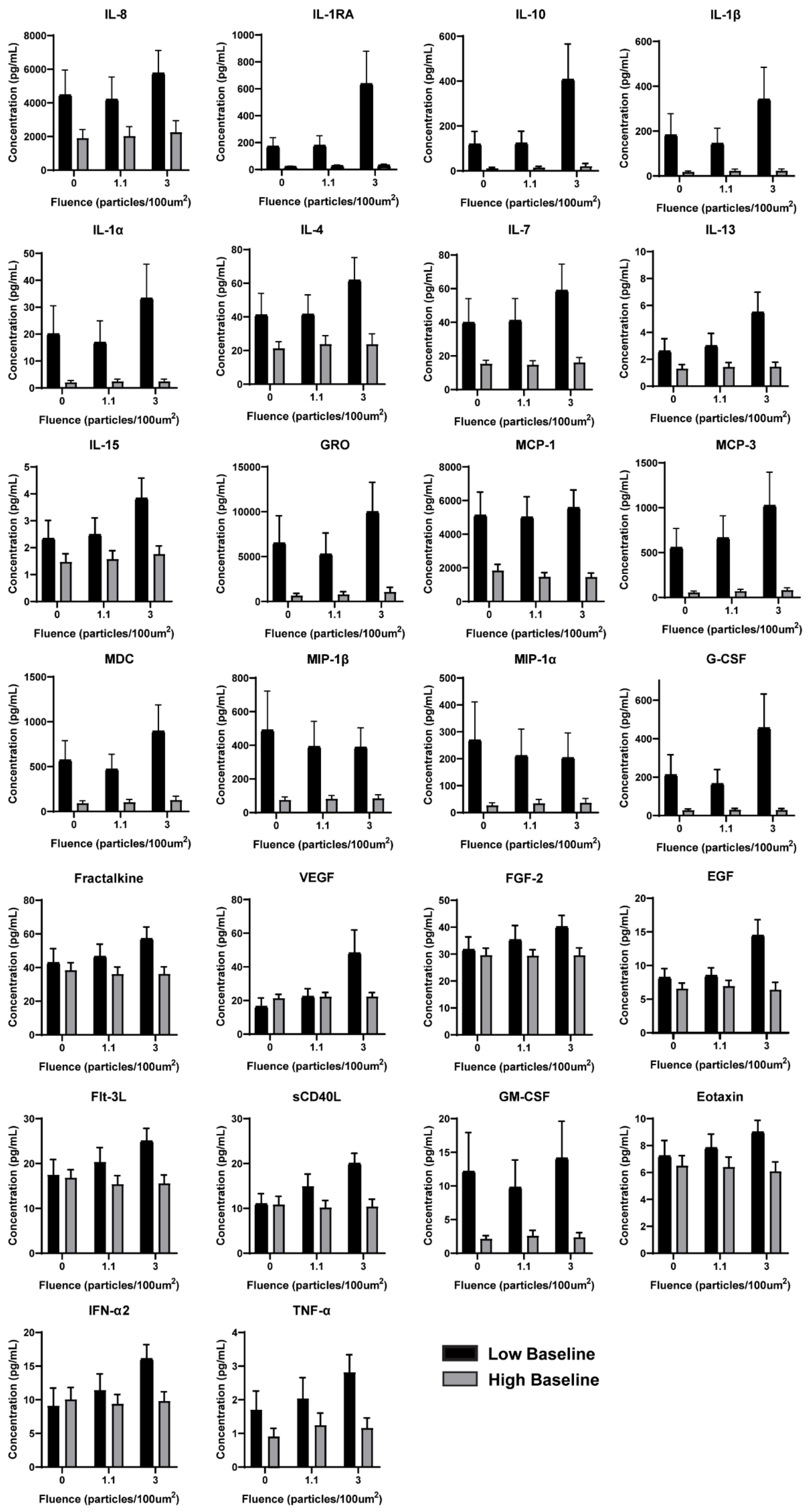
IL-8
IL-1RA
IL-10
IL-1β
IL-1α
IL-4
IL-7
IL-13
IL-15
GRO
MCP-1
MCP-3
MDC
MIP-1β
MIP-1α
G-CSF
Fractalkine
VEGF
FGF-2
EGF
Flt-3L
sCD40L
GM-CSF
Eotaxin
IFN-α2
TNF-α
Concentration (pg/mL)
Fluence (particles/100um^2)
0
1.1
3
Low Baseline
High Baseline

Table S1. Yield of ·OH and H_2O_2 production per Gray for each irradiation condition, Related to Figure 6.

Radiation	Energy (MeV/n)	LET (keV/µm)	·OH yield [(µmol/L)/Gy]	H_2O_2 yield [(µmol/L)/Gy]	·OH/H_2O_2 ratio
Gamma	-	-	0.167	0.0993	1.68
^{28}Si	350	63	0.119	0.0962	1.24
^{40}Ar	350	104	0.100	0.0983	1.02
^{56}Fe	600	170	0.0794	0.103	0.78
